# Shaping microbiology for 75 years: highlights of research published in *Microbiology*. Part 2 - Communities and evolution

**DOI:** 10.1099/mic.0.001357

**Published:** 2023-06-28

**Authors:** Michael Brockhurst, Jennifer Cavet, Stephen P. Diggle, David Grainger, Riccardo Mangenelli, Hana Sychrova, Isabel Martin-Verstraete, Martin Welch, Tracy Palmer, Gavin H. Thomas

**Affiliations:** ^1^​ Division of Evolution, Infection and Genomics, University of Manchester, Michael Smith Building, Dover Street, Manchester M13 9PT, UK; ^2^​ Lydia Becker Institute of Immunology and Inflammation, School of Biological Sciences, Faculty of Biology Medicine and Health, University of Manchester, Manchester M13 9PT, UK; ^3^​ Center for Microbial Dynamics & Infection, School of Biological Sciences, Georgia Institute of Technology, Atlanta, GA, USA; ^4^​ School of Biosciences, University of Birmingham, Edgbaston, Birmingham B15 2TT, UK; ^5^​ Department of Molecular Medicine, University of Padova, 35121 Padova, Italy; ^6^​ Institute of Physiology of the Czech Academy of Sciences, Laboratory of Membrane Transport, 14200 Prague 4, Czech Republic; ^7^​ Institut Universitaire de France, Paris, France; ^8^​ Department of Biochemistry, University of Cambridge, Cambridge, UK; ^9^​ Microbes in Health and Disease Theme, Newcastle University Biosciences Institute, Newcastle University, Newcastle upon Tyne, NE2 4HH, UK; ^10^​ Department of Biology, University of York, Wentworth Way, York, UK

## Introduction

In the first part of this overview of landmark papers published in the first 75 years of the journal *Microbiology*, we covered papers regarding bacterial growth, physiology and behaviour, and in this second part we now turn to papers more generally studying more ecological and evolutionary aspects of microbial life.

## Living together – microbes in their natural environment

Most biological niches contain a diverse collection of microbes. Working out ‘what is there’ and being able to tell the difference between them, i.e. microbial classification, is the topic of several seminal works. The first was a paper from the group of Stanier, who provided a classification scheme for the cyanobacteria, which, before the days of molecular fingerprinting, was hugely useful and widely adopted [1]. While classification has now improved due to genome sequencing, there was a period when rapid identification of bacteria using non-genetic methods was highly desirable. Addressing this, a paper from Goodacre and colleagues outlined a very different approach using spectrometry methods to produce organismal ‘fingerprints’. Fingerprints were created for all the known bacterial species linked to urinary tract infections. More pragmatically, the team showed that they could take a clinical sample from a patient with a UTI, pass it through their workflow, and rapidly identify the species present [2].

Once microbiologists had figured out what microbes are present in a particular niche and how to tell them apart, they realised that microbes interact with each other and have complex social lives involving both cooperative and competitive behaviours. The study of sociomicrobiology is gaining increasing relevance due to the great current interest in microbiomes and their impact on hosts. Recently, in the journal we created a new topic area of microbial interactions and communities to specifically capture this rapidly growing research area.

One important way bacteria communicate with each other is through the process of quorum sensing (QS), a research area that has developed rapidly since the early 1990s. QS involves the production of small diffusible signalling molecules by cells, that are released into the environment. When taken up by neighbouring cells, they stimulate the production of a number of shared QS-regulated factors. These include proteases, exopolymers and surfactants. By controlling such factors, QS can regulate environmental colonization, persistence and pathogenicity. While QS signals can now be detected in bacterial species using HPLC and mass-spectrometry methods, the field was initially facilitated by the construction of QS biosensors that used thin-layer chromatography (TLC). An early such QS biosensor was described in a highly cited *Microbiology* article from 1997 [3]. Here the researchers exploited a transposon mutant of *Chromobacterium violaceum* called CV026, which does not make QS signals or the QS-regulated purple pigment violacein. The authors found that CV026 responds to N-acyl homoserine lactone (AHL) molecules with carbon chains ranging from C_4_ to C_8_ in length by producing violacein, easily detected using TLC and CV026 became a key tool for many labs and it is still being used successfully today.

Understanding the role of QS in different organisms is key to understanding why such behaviours evolve. *P. aeruginosa* colonizes the cystic fibrosis (CF) lung by forming antibiotic-resistant biofilms in the airways, resulting in often untreatable chronic infection. Another highly cited *Microbiology* article published in 2005 examined the role of QS on antimicrobial tolerance in *P. aeruginosa* [4]. In this study, the authors showed that by using QS-inhibitory molecules, biofilms grown *in vitro* were made more sensitive to the antibiotic tobramycin, hydrogen peroxide and were more readily phagocytosed by polymorphonuclear leukocytes. This suggested that by combining QS inhibitory drugs with antimicrobials, biofilms in the CF lung could be more easily disrupted and cleared. However, the authors also made another important observation. They showed that QS mutants *in vivo* actually provoked a higher degree of inflammation. This finding has increased relevance today, because we now know that QS mutants are frequently isolated from CF lung infections, and they may be a cause of more rapid decline in lung function. The concept of QS inhibitory molecules is the topic of another highly cited paper in *Microbiology*, but here illustrating a different concept of cross-kingdom microbial interactions, as halogenated furanones produced by the red algae *Delisea pulchra* were demonstrated to interfere with the quorum-sensing cascade of the bacterium *Vibrio fischeri* [5], an early example of a principle now seen in many contexts as our understanding of complex communities increases.

A final related paper looked at what regulates production of one of the key biofilm matrix components, which is extracellular (secreted) DNA. Remarkably, this also seems to be under QS control. In this paper, Tolker-Nielsen’s team discovered that in the presence of high-iron concentrations, DNA release by *P. aeruginosa* is depressed (and consequently, biofilm stability is diminished) [6]. A possible mechanistic explanation for these observations is hinted at by the finding that *pqs* gene expression, which is known to be required for optimal DNA release, was also diminished under high-iron conditions. This paper is particularly interesting as it links metals, quorum sensing and biofilms in this important pathogen.

As well as secreting QS molecules in their environment, microbes also can export chemicals to kill competitors directly or that help them to acquire nutrients, or more importantly stop other organisms from capturing them. Over the years, many papers have been published in the journal on the discovery, characterization and production of diverse antibiotics, but one paper in particular made the highly cited list. This reported cloning of the biosynthetic cluster for the important front-line antibiotic daptomycin, relevant in the treatment of methicillin-resistant *Staphylococcus aureus* and vancomycin-resistant enterococci [7]. Daptomycin is a natural product produced by the actinomycete *Streptomyces roseosporus *, which causes permeabilization and depolarization of the bacterial cytoplasmic membrane. The method used to identify the gene cluster was the creation of a bacterial artificial chromosome (BAC) library containing fragments of the *S. roseosporus* genome and the identification of a BAC that could direct the synthesis of daptomycin when introduced into the heterologous host *Streptomyces lividans* [7]. Sequencing of this BAC revealed a large gene cluster over 100 kb long, from which the biosynthetic pathway was predicted, paving the way for the genetic modification of daptomycin peptide assembly to achieve greater efficiency, and for the generation of new derivatives that may have enhanced antibacterial activity.

Another important paper was the characterization of the fluorescent pigments known to be secreted by the ‘fluorescent pseudomonads’, that include the well-known pathogen *P. aeruginosa*, as well as *P. putida*, *P. fluorescens* (the principal protagonist in this paper) and *P. syringae*. These species are so-called because they secrete bright blue-green pigments when cultured, which had been known from the nineteenth century and later had been termed ‘pyoverdine’ (the pyo- prefix meaning ‘pus’, and dating back to the time when *P. aeruginosa* was most commonly associated with the blue-green pus issuing from wound infections). However, and in spite of its distinctive appearance, the physiological function of pyoverdine was not clear, even by the late 1970s. Enter Meyer and Abdallah [8], who showed that iron-limited *P. fluorescens* cultures overproduce the pigment, facilitating its chromatographic purification from spent culture supernatants. This, in turn, enabled determination of the molecular weight, spectral properties and gargantuan stability constant (*K*=1032!) of the Fe^3+^-pyoverdine complex. Based on these findings, Meyer and Abdallah correctly inferred that pyoverdine is a siderophore, laying the groundwork for rapid progress in the field over the subsequent decades.

Coming back to metals, which we discussed in the context of respiratory enzymes and homeostasis in part 1 of this series, there is another aspect of their interaction with microbes in the area of geomicrobiology. This was covered in an influential review article by mycologist Geoff Gadd [9], who was recently awarded the Microbiology Society’s Marjory Stephenson Prize to reflect his major contribution to this field. Microbes currently play key roles in transforming organic and inorganic substrates in the biosphere, and over much longer timer periods have catalysed the biogeochemical cycling of elements and the chemical transformations of metals and minerals. This interesting area is one that has the potential to grow further, particularly in biotechnological applications where metal recovery, for example, from waste produced by electronic devices, and bioremediation will drive a heightened awareness of metal–microbe interactions.

## Genetics and evolution

At the foundation of the journal molecular genetic tools were essentially non-existent. The development of such methods has been a triumph of twentieth century microbiology, with Nobel prizes for numerous leading proponents. In the pages of *Microbiology* important methodological breakthroughs have been published for many organisms. Two particularly important papers on methods for the pathogen *Mycobacterium tuberculosis* (Mtb) stand out. Despite Mtb being a formidable and ancient human pathogen, its study before the 1990s had been limited due to both the perception in the Western world that tuberculosis was not a real problem anymore, and the practical difficulties of working with this organism in the laboratory. These difficulties are numerous: Mtb needs a high level of biological containment requiring special facilities, is very slow to grow (it takes about 3 weeks to see a colony), and, in that period, genetic manipulation was very difficult due to the high frequency of illegitimate recombination. In the classic first paper, Tanya Parish, a previous Editor-in-Chief of the journal, with Neil Stoker, described a two-step method based on suicide plasmids to generate unmarked mutants [10]. Although highly efficient, the nature of the two-step process, where a plasmid was first introduced and recombined onto the chromosome, followed by a second counter-selection to remove the remnant of the plasmid, was slow. Two years later, Stoyan Bardarov and colleagues from Bill Jacobs’ laboratory, described a specialized bacteriophage transduction method to produce marked and unmarked targeted gene disruptions in *M. tuberculosis* [11]. This was based on the construction of a temperature-sensitive mycobacteriophage containing the gene of interest disrupted by a selectable marker, which was able to deliver this construct to *M. tuberculosis* with very high efficiency. Later on, it was possible to remove the selectable marker using the gamma-delta resolvase. While the method was faster than that of Parish and Stoker, it had a lower success rate due to problems of stability of the mycobacteriophage or incorrect integration of the transducing construct in the Mtb genome. However, the two methods are complementary, and remain widely used in the Mtb community.

Sticking with Mtb and methods, another highly cited paper from a London-based research team at St George’s Hospital Medical School, was published in *Microbiology* looking at the transcriptional response to life in the human lung [12]. Using a combination of genetics and the DNA microarrays, which were new at the time, they dissected the heat-shock response of Mtb, which is important when the bacteria grow inside human cells. The paper focused on two arms of the regulatory response to heat and became a citation hit, both for its direct insight into Mtb infection biology and also due to the conservation of many of the identified stress responses in other bacteria.

Routine DNA sequencing has enabled the topic of molecular phylogenetic to provide unprecedented insight into the distribution and spread of genes and many studies of this type have appeared in the journal’s pages. Notably these have often focused upon Rhizobia, the nitrogen-fixing symbionts of legumes, which usually contain multiple replicons of highly variable size and number. Important early studies focused on key genes involved in nitrogen fixation and nodulation, many from researchers who had been associated with *Microbiology* Editor-in-Chief John Postgate’s Nitrogen Fixation Laboratory (NFL). Many of these studies revealed a fascinating disconnection between the evolutionary histories of the symbiosis genes and the chromosomal backbones of their bacterial hosts. Highly diverged taxa often contain very closely related *nod* and *nif* genes owing to the propensity for these key symbiosis genes to be encoded on mobile genetic elements, usually plasmids, that can be horizontally transferred between species [13].

As exemplified by Rhizobia, the horizontal transfer of genes between bacterial species often relies upon broad host-range plasmids. However, the ability of these plasmids to persist in bacteria in the absence of direct selection can vary dramatically between even closely related strains. Plasmid stability in bacterial populations depends upon the rates of plasmid segregation and conjugation, as well as fitness costs and the potential for these to be ameliorated by compensatory evolution. This was elegantly demonstrated in an comparative study from Eva Top’s group at the University of Idaho, USA, combining experiments with an IncP-1 plasmid with mathematical modelling to reveal the underlying mechanisms why plasmid stability varies so much [14].

A related application of genome sequencing has led to a renaissance in studies of microbial evolution, and this has recently been clearly designated as a new topic area in *Microbiology* since 2021. The journal has published important research, where sequencing has allowed immediate tracking of evolutionary change, including an article recently published in this new topic area by a pioneer of microbial evolutionary biology and designer of the long-term evolution experiment (LTEE), Richard Lenski [15].

One system where observational studies of evolutionary adaptation has been particularly insightful is during pathogenesis, most notably the chronic *P. aeruginosa* airway infections associated with individuals living with cystic fibrosis. One such study used genome sequencing to track the emergence of key phenotypes associated with chronic infection over time in multiple patients. This revealed that mucoidy and loss of quorum sensing evolved in both hypermutator and non-hypermutator backgrounds, whereas the eventual emergence of high-level antimicrobial resistance was associated with hypermutability [16]. The apparent success of hypermutators arises because these alleles hitchhike on adaptive mutations, such as those providing antimicrobial resistance, as these are swept to high frequency by positive selection. Over the longer term, however, hypermutators pay the price for their elevated mutation rates in terms of reduced ability to compete and colonize new environments as they accumulate more and more damaging mutations [17].

No tour of microbial evolution research would be complete without some experimental evolution and *Microbiology* has published its fair share of classic studies. Evolutionary innovations are difficult to study in nature but sometimes arise in the lab, offering a rare glimpse into the origins of a new traits and in fact, Frederick Twort at the Brown Institute in London was one of the first to observe this in 1907 when he found that he could experimentally evolve new sugar utilizing phenotypes in *Salmonella* sp. in the lab [18]. In modern times, one very well-studied system has occupied many pages in the journal, that being the evolution of biofilm-forming so-called ‘wrinkly spreaders’ that evolve within days in static broth cultures of *P. fluorescens*, enabling these novel genotypes to occupy a new niche at the air–liquid interface. A detailed comparison of wrinkly spreader genotypes by Spiers and Rainey shows that the floating biofilm mat they create is primarily due to overexpression of cellulose fibres, but also relies on attachment and interactions with the LPS for its strength, revealing the complexity of evolutionary adaptations possible even in the simplest of laboratory settings [19].

## Conclusions

In reviewing over two articles some of our most impactful papers from the first 75 years of the journal, one can see how the subject of microbiology has changed. In many cases, this is due to technological and methodological developments that have enabled researchers to tackle important problems in new ways. For example, our research has been shaped by DNA sequencing, and publication of the first complete bacterial genome, *Haemophilus influenzae* Rd in 1995, marked a major transition in the last quarter of the journal's existence, and in fact led to the creation of a new journal *Microbial Genomics* by the Microbiology Society.

What might we look forward to for our 100 year anniversary? One hates to make predictions, but luckily we can draw on a beautifully outlined personal history of the subject by Roberto Kolter [20] where he posits that ‘it’s all environmental microbiology’. What Roberto is ealluding to here is that we can build on our extensive knowledge of individual species and understanding of the composition of biological niches, to work towards holistic understanding of how microbes function in full ecosystems from our guts to our skin, to the soil, the sea, the sky and so on. While this must be our aspiration as biologists, we will be aided by new technologies that will allow us to look progressively deeper into living microbial cells, combined with the power of artificial intelligence (AI), where we have already seen the enormous impact of machine learning on structural biology through AlphaFold and similar structure prediction tools. One can imagine computational models of microbial cells that can describe all the molecular interactions that are occuring and measure their outcomes over progressively longer timescale, perhaps then enabling these mechanistic models of individual cells to be added into larger scale biological networks in truly systems biology-type approaches. It is always an exciting time to be a microbiologist!

One thing we know for certain is that our 76th year started with a major transition to open access publishing, marking another milestone for the journal and opening up our published research to all readers. To support this important transition, we need you to continue to publish your research with us, through our Publish and Read agreements, to keep the journal strong on its journey through the next quarter century of microbiology.
